# Mineral disorders after hematopoietic stem cell transplantation in patients receiving parenteral nutrition

**DOI:** 10.1016/j.htct.2026.106353

**Published:** 2026-03-21

**Authors:** Taís Daiene Russo Hortencio, Julia Daudt de Faro Salamonde, Alice Missagia de Mattos Springer, Afonso Celso Vigorito, Vitória de Andrade Mendonça, Roberto José Negrão Nogueira

**Affiliations:** a Faculdade de Ciências Médicas, Universidade Estadual de Campinas – Unicamp. Rua Tessália Vieira de Camargo, 126, Cidade Universitária “Zeferino Vaz”, Barão Geraldo, Campinas, São Paulo, CEP: 13083-887, Brasil; b Faculdade de Medicina São Leopoldo Mandic. Rua Dr. José Rocha Junqueira, 13, Ponte Preta, Campinas, São Paulo, CEP: 13045-755, Brasil; c Hemocentro da Universidade Estadual de Campinas, SP, Brasil; d Faculdade de Ciências Médicas, Universidade Estadual de Campinas – Unicamp. Rua Tessália Vieira de Camargo, 126, Cidade Universitária “Zeferino Vaz”, Barão Geraldo, Campinas, São Paulo, CEP: 13083-887, Brasil; e Departamento de Medicina Interna, Faculdade de Ciências Médicas, Universidade Estadual de Campinas – Unicamp. Rua Tessália Vieira de Camargo, 126, Cidade Universitária “Zeferino Vaz”, Barão Geraldo, Campinas, São Paulo, CEP: 13083-887, Brasil

**Keywords:** Parenteral nutrition, Hematopoietic stem cell transplantation, Hypophosphatemia, Hypokalemia, Hypomagnesemia

## Abstract

**Background:**

Parenteral nutrition is a critical therapeutic approach for patients undergoing hematopoietic stem cell transplantation. However, parenteral nutrition is associated with complications such as mineral disorders. This study evaluated the prevalence of hypophosphatemia, hypokalemia, and hypomagnesemia in adult transplant patients before and within the first 24 days of parenteral nutrition infusion.

**Patients and Methods:**

This retrospective cohort study included patients who underwent hematopoietic stem cell transplantation and required parenteral nutrition at a public quaternary hospital in Brazil between January 2012 and January 2022. Patients were categorized based on indications for parenteral nutrition and specific diagnoses. Laboratory monitoring of phosphorus, potassium, and magnesium levels was performed at predefined intervals during parenteral nutrition infusion, starting at 72 h and during subsequent 4-day intervals until engraftment. Hypophosphatemia, hypokalemia, and hypomagnesemia were defined as levels below established reference values. Descriptive analyses were applied, and the chi-square test, crude odds ratios, and Fisher's exact test were used to compare categorical variables between the periods.

**Results:**

The study revealed varying prevalences of hypophosphatemia (21.1–40.3 %), hypokalemia (10.5–31.2 %), and hypomagnesemia (50.0–62.3 %) among the 77 patients included in the study. Severe mineral disorders were observed in only a small proportion of patients.

**Conclusion:**

Patients undergoing hematopoietic stem cell transplantation had a high prevalence of mineral depletion throughout the engraftment period.

## Introduction

Side effects related to hematopoietic stem cell transplantation (HSCT) conditioning can lead to severe mucositis, impair oral intake, chewing, and swallowing, and suggest a need of special nutritional support for these patients [1]. Mucositis lesions are common occurrences in oncology patients, with a reported incidence of 40 % in those receiving chemotherapy and 76 % in patients undergoing hematopoietic stem cell transplantation (HSCT). This high prevalence frequently leads to poor tolerance of oral or enteral feeding routes [2] and so the parenteral route is preferred.

Parenteral nutrition (PN), although a vital lifeline for patients undergoing hematological recovery, is associated with numerous complications with mineral disorders having emerged as a prominent concern [3]. Mineral disorders in patients receiving PN predominantly arise from two key factors. First, patients undergoing HSCT often require complex regimens of medications to manage their underlying conditions [4]. These medications typically include immunosuppressants to prevent graft-versus-host disease (GvHD), antibiotics to prevent infections, and other drugs to manage post-transplant complications. Unfortunately, these drugs can significantly disrupt the delicate balance of essential electrolytes in the body, leading to imbalances in minerals such as potassium, magnesium, and phosphorus. These imbalances can have far-reaching consequences, affecting the patient's overall health and potentially hindering the success of the hematological recovery process [5]. The second factor contributing to mineral disorders in HSCT patients is the risk of Refeeding syndrome, particularly within the initial days after starting PN. Refeeding syndrome is a severe metabolic disturbance characterized by rapid shifts in mineral levels, particularly potassium, phosphorus, and magnesium, as the body adapts to increased nutritional intake after a period of malnutrition or fasting [6]. When HSCT patients, who are often in a compromised nutritional state before transplantation, begin PN abruptly, they are particularly susceptible to Refeeding syndrome. This condition can lead to various complications, including cardiac arrhythmias, respiratory distress, and neuromuscular disturbances [7]. The correct management of the mineral disorders in these vulnerable patients is paramount to ensuring successful hematological recovery while minimizing the potential complications associated with PN.

This study evaluated the prevalence of hypophosphatemia, hypokalemia, and hypomagnesemia before and within the first 24 days of PN infusion in adult patients who underwent HSCT.

## Methods

### Setting, study design, inclusion, and exclusion criteria

A retrospective historical cohort study was conducted of patients with malignant and non-malignant hematological diseases who underwent autologous or allogeneic HSCTs at a public quaternary hospital in Campinas, southeastern region of Brazil, over ten consecutive years. The study was approved by the local research ethics committee (#56610022.8.0000.5404).

Data from all patients who underwent HSCT and received PN were collected using a standardized form previously prepared for managing and monitoring PN patients. This form contains the total PN prescribed, details of oral and enteral intake, as well as clinical and laboratory data. All patients who underwent HSCT and required PN for at least 24 hours or until they began receiving full enteral or oral nutrition were included. Patients were classified according to their indications for PN, which included neutropenic colitis, GvHD, and grade III and IV mucositis. The data were filled in by the Nutritional Multidisciplinary Therapy Team (NMTT), composed of a dietitian, physician, nurse, and pharmacist involved in patient care. Forms with conflicting or inconsistent data were excluded.

### Laboratory monitoring

Laboratory monitoring was first performed before starting PN and followed a routine based on the current protocol for the laboratory monitoring of PN [8]. In the service, the metabolic assessment was performed immediately before PN and until the day before engraftment or death. The serum levels of phosphorus, potassium, and magnesium were obtained within the first 72 h (Period 1 [P1]), and for each subsequent 4-day period (Period 2 [P2]: Days 4–7; Period 3 [P3]: Days 8–11; Period 4 [P4]: Days 12–15; Period 5 [P5]: Days 16–19; and Period 6 [P6]: Days 20–24). Energy and protein levels were evaluated throughout the period.

Hypophosphatemia, hypomagnesemia, and hypokalemia were defined as levels below the established laboratory reference values, as follows: phosphorus 2.5–4.5 mg/dL, magnesium 1.32–2.14 mEq/L, potassium 3.5–5.1 mEq/L [9, 10]. A severely depleted mineral status was defined as a plasma concentration less than or equal to half the minimum reference level. In patients exhibiting severely depleted mineral levels, serum concentrations were individually corrected prior to the initiation of PN. This correction was achieved via intravenous infusion of the specific mineral at the highest possible dose, adhering to established guidelines and pharmacokinetic compatibility considerations [11]. Any new incidence of hypophosphatemia, hypokalemia, or hypomagnesemia observed during the P2−P6 period was considered an event.

### Anthropometric assessment

In the service, weight and height of all patients were measured. The technique adopted for collecting measurements was based on recommendations proposed by the Anthropometric Standardization Reference Manual [12]. Nutritional status was assessed according to the growth charts of the World Health Organization [13]. The measured weight was obtained using a digital electronic scale (Filizola®, São Paulo, Brazil), with a capacity of 150 kg and precision of 100 g, and the height was recorded using a wooden stadiometer with a precision of 0.1 cm, which was attached to a 200-cm-long flat vertical surface

### PN prescription and monitoring routine

Before the start of infusion, levels of minerals (sodium, potassium, ionic or total calcium, chloride, magnesium, and phosphorus), triglycerides, cholesterol, high-density lipoprotein (HDL), alanine aminotransferase, gamma-glutamyl transferase, hemoglobin, albumin, transthyretin, urea, creatinine, and glucose were measured in all patients. The mineral levels of potassium, magnesium, and phosphorus was measured three times a week in all the patients.

Prescriptions were formulated with the aid of the supplying pharmacy´s computer software, which performs pharmacokinetics analysis to ensure compatibility of the constituent components. PN was prescribed by two physicians.

The recommendations for energy-protein and the proportions of macronutrients, minerals, vitamins, and micronutrients were prescribed according to the patient's clinical condition and the guidelines established by the American Society for Parenteral and Enteral Nutrition (ASPEN) [14]. Subsequent adjustments, where required, were made based on clinical, nutritional, and laboratory monitoring.

The initial standardized solutions, the values of phosphorus, potassium, and magnesium used for PN infusion, were those recommended by current guidelines (phosphorus: 7–10 mmol/1000 kcal/day, potassium: 1–2 mEq/kg/day, and magnesium: 8–28 mEq/day) [8,10, 11, 12, 13, 14].

The amount of energy, protein, and non-protein calorie/gram of nitrogen ratio were evaluated in P1–P5 until engraftment, defined as a neutrophil count ≥0.5 × 10^9^ L for two consecutive days and platelet count ≥20 × 10^9^ L for three consecutive days without transfusion for one week.

### Energy calculation of the accepted nutrition

The total energy provided by PN was calculated based on the macronutrient content, using the following conversion factors: 4 kcal/g for amino acids, 3.4 kcal/g for carbohydrates, and 10 kcal/g for lipids. The resulting values were compared against the recommended daily requirements of 23–30 kcal/kg for energy and 1–1.5 g/kg for protein [15]. Regarding the oral diet, acceptance was analyzed in the previous day’s collection chart.

### Statistical analysis

The chi-square test, crude odds ratios (ORs), and Fisher’s exact test were used to compare categorical variables between the groups. Confidence intervals for the proportions were calculated using the standard frequentist method. Analyses were performed using the Statistical Package for the Social Sciences version 16 (IBM Company, Chicago, IL, USA). Differences were considered statistically significant at a two-sided p-value <0.05.

## Results

A total of 77 adult patients who underwent HSCT were included in this study. The mean age of the patients was 46.4 years and 50.6 % were male. Most patients had myeloid malignancies (50.6 %) and lymphoid malignancies (44.2 %). Autologous HSCT transplantation was performed in 41.6 % of cases, human leukocyte antigen (HLA) matched related and unrelated HSCT in 50.6 % and 6.5 %, respectively, and HLA-mismatched related transplantation in 1.3 %. The conditioning regimen types included high doses without total-body irradiation (81.8 %), high doses with total-body irradiation (1.3 %), and reduced intensity (16.9 %). Platelet engraftment occurred at a median of 18 days (interquartile range [IQR]: 16–22 days), and neutrophil engraftment occurred at a median of 14 days (IQR: 11–17 days) (Table 1).

**Table 1:** Demographic and clinical data of adult patients receiving hematopoietic stem cell transplantation and parenteral nutrition.

Characteristic	n= 77
**Age** – years ± SD	46.4 ± 14.6
**Male -** n (%)	39 (50.6)
**Diagnosis at Transplant -** n (%)	
Myeloid malignancy	39 (50.6)
Lymphoid malignancy	34 (44.2)
Non-hematological malignancy - Others⁎	4 (5.19)
**Donor type -** n (%)	
Autologous	32 (41.6)
HLA-matched related	39 (50.6)
HLA-matched unrelated	5 (6.5)
HLA mismatched related	1 (1.3)
**Conditioning regimen types -** n (%)	
High dose	
Without total body irradiation	63 (81.8)
With total body irradiation	1 (1.3)
Reduced intensity	13 (16.9)
**Platelet engraftment** – days n (IQR)	18 (16-22)
**Neutrophils engraftment** – days n (IQR)	14 (11-17)

IQR: Interquartile range.

⁎ Others included aplastic anemia, amyloidosis, polycythemia, and neutropenia.

The mean BMI was 25.6 kg/m^2^ with 40.3 % of patients overweight and 13.0 % obese. Among the patients, 14.3 % died with PN administered for a mean duration of 1.4 days. The main reason for PN indication was grade IV mucositis (88.3 %) (Table 2).

**Table 2:** Nutritional status, reasons for PN, and mucositis severity in patients receiving hematopoietic stem cell transplantation and parenteral nutrition.

Clinical data	Values
**BMI** - (kg/m^2^ ± SD)	25.6 ± 3.7
**Nutritional status -** n (%)	
Underweight	4 (5.2)
Normal range	32 (41.6)
Overweight	3 (40.3)
Obese	10 (13.0)
**Mortality -** n (%)	22 (14.3)
**Time of PN**	1 (1.4)
**Reasons for PN -** n (%)	
Neutropenic colitis	1 (1.3)
GvHD	3 (3.9)
Mucositis level III	5 (6.5)
Mucositis level IV	68 (88.3)

PN: Parenteral Nutrition; BMI: Body Mass Index; GvHD: Graft-versus-host disease.

Energy intake per day varied across different periods, ranging from 23.1–36.8 kcal/kg. Protein intake per day also varied across the study period, ranging from 0.96–1.62 g/kg. The non-protein calorie/g of the nitrogen ratio was also evaluated, with values below and above the recommended target values observed during different periods (Table 3).

**Table 3:** Nutritional intake by oral and parenteral prescription in adult patients receiving hematopoietic stem cell transplantation and parenteral nutrition.

Variable	P1	P2	P3	P4	P5	P6
	Median (p25-p75)	Median (p25-p75)	Median (p25-p75)	Median (p25-p75)	Median (p25-p75)	Median (p25-p75)
Energy per day -kcal/kg	23.1 (15.9-28.0)	35.3 (26.5-42.0)	34.8 (27.3. 40.4)	36.8 (29.3-42.0)	35.9 (22.5-41.3)	27.9 (27.9-27.9)
Energy requirement (minimum) - %^b^	92.5 (63.4-112.1)	141 (106.0-168.0)	139.4 (109.4-161.7)	147.5 (117.2-168.0)	143.5 (90.0-165.4)	111.6 (111.6-111.6)
Energy requirement (maximum) - %^c^	77.0 (53.2-93.4)	117.7 (88.3-140.2)	116.1 (91.2-134.7)	122.9 (97.7-140.0)	119.7 (75.0-137.9)	93.0 (93.0-93.0)
Protein per day, g/kg	0.96 (0.69-1.14)	1.57 (1.25-1.87)	1.62 (1.40-1.93)	1.61 (1.43-1.97)	1.59 (1.22-1.79)	1.02 (1.02-1.02)
Protein requirement (minimum) - %^d^	96.1 (69.8-114.9)	157.6 (125.9-187.5)	162.4 (140.3-193.4)	162.4 (140.9-197.7)	159.7 (122.5-179.1)	102.1 (102.1-102.1)
Protein requirement (maximum) - %^e^	48.0 (34.9-57.4)	78.8 (62.9-93.7)	81.2 (70.1-96.7)	81.2 (71.9-98.8)	79.8 (61.5-89.5)	51.1 (51.1-51.1)

Variables were compared with the Mann-Whitney *U* test or Fisher’s exact test as appropriate.

b,c Energy adequacy was calculated using reference values of 25 kcal/kg/day for minimum requirements and 30 kcal/kg/day for maximum requirements.

d,e Protein adequacy was calculated using reference values of 1.0 g/kg/day for minimum requirements and 2.0 g/kg/day for maximum requirements.

Table 4 provides information on the occurrence of mineral disorders during the HSCT detailed by period (P1–P6). The prevalence of hypophosphatemia ranged from 21.1–40.3 % with the highest occurrence observed in P2. Hypokalemia was observed in 10.5–31.2 % of patients with the highest occurrence in P1. The incidence of hypomagnesemia ranged from 50.0–62.3 % with the highest incidence in P1. Severe hypophosphatemia events were observed in 1.6 % of patients in P2. Severe hypokalemia was observed in 2.6 % and 2.7 % of patients in P2 and P4, respectively. Severe hypomagnesemia occurred in 1.3 % and 1.8 % of patients in P1 and P3, respectively.

**Table 4:** Mineral disorders in adult patients receiving hematopoietic stem cell transplantation and parenteral nutrition.

Variable	Period 1 (n = 77)	Period 2 Days 1-4 (n = 67)	Period 3 Days 4-7 (n = 55)	Period 4 Days 8-11 (n = 36)	Period 5 Days 12-15 (n = 19)	Period 6 Days 16-19 (n = 0)
	n (%)	n (%)	n (%)	n (%)	n (%)	n (%)
Hypophosphatemia	22 (28.6)	27 (40.3)	20 (36.4)	12 (33.3)	4 (21.1)	0 (0.0)
Hypokalemia	24 (31.2)	18 (26.9)	10 (18.2)	10 (27.7)	2 (10.5)	0 (0.0)
Hypomagnesaemia	48 (62.3)	38 (56.7)	34 (61.8)	18 (50.0)	11 (57.9)	0 (0.0)

## Discussion

There was a high number of mild intracellular mineral disorders. Disorders of intracellular minerals are noteworthy for their frequency. Hypokalemia, hypomagnesemia, and hypophosphatemia have been reported during the PN period; this phenomenon might be explained by the release of insulin associated with the parenteral energy infusion [16]. However, both hypokalemia and hypomagnesemia occurred in almost half of the samples during the first four days of PN when the amount of energy offered was still low; therefore, it is likely that, in addition to refeeding, there was tubulopathy caused by pre-transplant chemotherapy. A study of patients undergoing HSCT observed an incidence of acute kidney injury of 64 %, occurring more frequently in those with hypoalbuminemia [17].

Electrolyte disturbances are often associated with patients taking antineoplastic agents; this can lead to serious or fatal clinical manifestations [18]. In this study, hypokalemia and hypomagnesemia were found in 31.2 % and 62.3 % of patients, respectively. These disorders frequently occurred concomitantly, which may be explained by the association between proximal tubule damage and the blockage of the TRPM6/EGF/EGFR pathway. This blockage impairs magnesium reabsorption in the distal tubule, thereby preventing the maintenance of normal serum magnesium and calcium levels [18]. Although drug-induced tubulopathy can explain hypokalemia and hypomagnesemia, patients undergoing HSCT often experience a period of low food intake and reduced lean body mass due to processes related to the neoplasia. An underlying inflammatory process is present, characterized by anorexia and insufficient energy and protein intake. This condition is further exacerbated by the metabolic redirection of protein stores toward the synthesis of pro-inflammatory factors [19]. Thus, these factors may have contributed to hypokalemia and hypomagnesemia associated with drug tubulopathy. However, considering the prevalence of hypokalemia and hypomagnesemia during the first week of PN, it is not possible to separate the effects of tubulopathy from what can be attributed to refeeding caused mainly by PN [3].

Phosphorus is another intracellular mineral and is, therefore, characteristically depleted in the Refeeding syndrome. In the current study, the drop in this anion was most evident after the fourth day of PN (40.3 % of patients). Therefore, it can be understood that the fact that phosphorus had a more significant drop in plasma levels during the second period of PN is probably attributable to cellular Theft syndrome, given that the amount of energy administered, although without hyperalimentation, was already increased during this period [3,20]

Although there was a high prevalence of mineral depletion throughout the study, few patients had severe levels of mineral depletion. This can be directly attributed to the fact that rigorous monitoring of both the nutritional and metabolic issues occurred daily, owing to the presence of an NMTT. Notably, the patients managed to achieve their target, indicating the active involvement of the team [21].

In the 77 patients studied, Grade IV mucositis was present in 88.3 % of the cases as expected given that the patients had to receive PN as their main source of nutrition. Patients with Grade IV mucositis experience significant discomfort when eating because of intense inflammation and ulceration with necrotic lesions; however, the use of enteral tubes remains controversial [22].

In the present cohort, the use of PN was effective in meeting energy and protein needs with the amount of energy offered reaching 36.8 kcal/kg/day, and protein reaching 1.62 g/kg/day. It has been previously reported that the amount of energy needed after HSCT increases by 30–50 %, and the amount of protein should reach approximately 1.5 g/kg/day [23]. This was only possible because the place where this research was conducted by the NMTT was prepared to prescribe customized PN, with the possibility of optimizing the number of amino acids prescribed. Studies have shown that the presence of an NMTT facilitates this type of approach [24,25].

### Study limitations

Given this was a retrospective cohort, the original data were recorded by many different individuals as opposed to a sole researcher. However, to attenuate this aspect, care was taken to remove any inconsistent records. Moreover, all the charts obtained were checked by a second member of the team.

## Conclusion

Patients undergoing HSCT are at risk of developing mineral disturbances during the engraftment period. Although these factors diminish over time, disorders remain prevalent in all periods. Therefore, rigorous daily monitoring of these patients by a team specializing in PN is warranted.

## Financial disclosure statement

This research did not receive any specific grant from funding agencies in the public, commercial, or not-for-profit sectors.

## Conflicts of interest

None.
